# Sex‐specific predictive value of reticulated platelets in coronary artery disease: A systematic review and meta‐analysis

**DOI:** 10.1111/eci.70078

**Published:** 2025-05-19

**Authors:** Sebastien Elvinger, Stephanie G. Kuehne, Andrea Patrignani, Maximilian Tscharre, Matthias Freynhofer, Leor Perl, Ran Kornowski, Francesca Cesari, Rossella Marcucci, Laura Novelli, Isabell Bernlochner, Philip W. Raake, Mauro Chiarito, Dario Bongiovanni

**Affiliations:** ^1^ Department of Internal Medicine I, Cardiology University Hospital Augsburg, University of Augsburg Augsburg Germany; ^2^ Department of Cardiovascular Medicine Humanitas Clinical and Research Center IRCCS and Humanitas University Rozzano Milan Italy; ^3^ Department of Internal Medicine with Cardiology, Nephrology and Intensive Care Medicine Universitätsklinikum Wiener Neustadt Wiener Neustadt Austria; ^4^ Department of Medicine Faculty of Medicine and Dentistry, Danube Private University Krems Austria; ^5^ 3rd Medical Department, Cardiology and Intensive Care Medicine Clinic Ottakring Vienna Austria; ^6^ The Cardiovascular Division Beilinson Hospital, Rabin Medical Center, Petach Tikva and the Faculty of Medical and Health Sciences, Tel Aviv University Tel Aviv Israel; ^7^ Department of Experimental and Clinical Medicine University of Florence Florence Italy; ^8^ Atherothrombotic Diseases Centre Careggi University Hospital Florence Italy; ^9^ Department of Internal Medicine I School of Medicine, University Hospital Rechts der Isar Munich Germany

**Keywords:** cardiovascular disease, immature platelets, reticulated platelets, sex differences

## Abstract

**Background:**

Platelets play a crucial role in immune responses and haemostasis. Among them, reticulated platelets (RPs) have gathered attention for their association with prothrombotic states and as a potential biomarker for cardiovascular events. However, the sex‐specific prognostic value of RPs remains underexplored.

**Objective:**

This study aimed to systematically review and analyse sex‐specific differences in the prognostic role of RPs in cardiovascular disease.

**Methods:**

We conducted a comprehensive search on studies that reported patient outcomes related to RPs. Study authors were contacted to provide sex‐specific patient‐level data. Two studies were excluded due to data unavailability. The primary endpoint was major adverse cardiovascular and cerebrovascular events (MACCE). Secondary endpoints included cardiovascular death, myocardial infarction, stroke, urgent revascularization, and bleeding incidents. All outcomes were stratified by sex.

**Results:**

The analysis included 5 studies, reporting outcomes in 1835 patients (527 females and 1308 males). RPs are a significant predictor of MACCE independently of sex males (OR 1.99 [95% CI 1.3, 3.05; *I* ^2^ = 29%]), females (2.29 [95% CI 1.31, 3.99; *I* ^2^ = 10%]). For cardiovascular death RPs were predictive in females (OR 3.29 [95% CI 1.69, 6.40] *I* ^2^ = .83%) and showed a trend toward significance in males (OR 2.19 95% CI [.98, 4.9] *I* ^2^ = 42.72%). No sex‐specific differences were observed in all other secondary endpoints.

**Conclusion:**

RPs significantly predict MACCE in cardiovascular disease independently from sex and may have a stronger association with cardiovascular death in females. Further research is needed to explore the sex‐specific mechanisms of RPs' prognostic value.

## Graphical abstract

This meta‐analysis of 1835 patients (527 females, 1308 males) shows that elevated RPs are significant predictors of MACCE in both sexes (males: OR 1.99, 95% CI 1.3–3.05; females: OR 2.29, 95% CI 1.31–3.99). Elevated RPs are more strongly associated with cardiovascular death in females (OR 3.29, 95% CI 1.69–6.40) compared to males (OR 2.19, 95% CI .98–4.90), suggesting potential sex‐specific differences. No significant sex‐based differences were observed for secondary endpoints, including myocardial infarction, stroke, urgent revascularization, and bleeding.

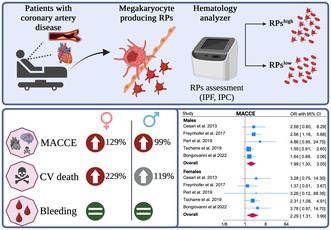

## 1 INTRODUCTION

Platelets, traditionally recognized for their pivotal role in vascular haemostasis, have emerged as key contributors to a spectrum of physiological and pathophysiological processes, including immune responses and tumour progression.^1^, ^2^ Beyond their well‐documented functions in thrombogenesis, recent advances in platelet research have revealed a remarkable complexity within these anucleated cells, particularly their capability for RNA processing and translation.^3^ Variations in size, surface receptors expression, and granule content among platelets suggest a sophisticated level of functional specialization that could extend well beyond haemostasis.^3^

Immature or reticulated platelets (RPs) are young, large, and RNA‐rich platelets, known for their hyperreactivity. This prothrombotic phenotype may partly reflect their recent release from bone marrow megakaryocytes.^4^ However, selective RNA and protein sorting during thrombopoiesis at the megakaryocyte level may also contribute to this heterogeneity, suggesting that platelet function is not solely determined by aging.^5^

Interest in RPs has recently intensified, driven by their association with prothrombotic states and significant correlations with adverse clinical outcomes in various diseases.^6^, ^7^ In particular, elevated levels of RPs have been linked to acute cardiovascular events and poor long‐term outcomes even in patients treated with optimal antiplatelet therapy.^6^, ^7^

Despite their potential as biomarkers, the prognostic value of RPs across different patient populations, particularly by sex, remains underexplored. Sex‐specific responses in cardiovascular and systemic diseases have been increasingly recognized,^8^, ^9^ yet females remain underrepresented in clinical and preclinical studies.^9^ We previously reported in a large meta‐analysis that elevated RPs are significantly associated with an increased risk of cardiovascular events and cardiovascular death.^6^ Building on these findings, this study aims to systematically review and analyse the predictive role of RPs in adverse cardiovascular events, stratified by sex, using the totality of existing evidence.

## 2 MATERIALS AND METHODS

### 2.1 Literature Search

**FIGURE 1:**
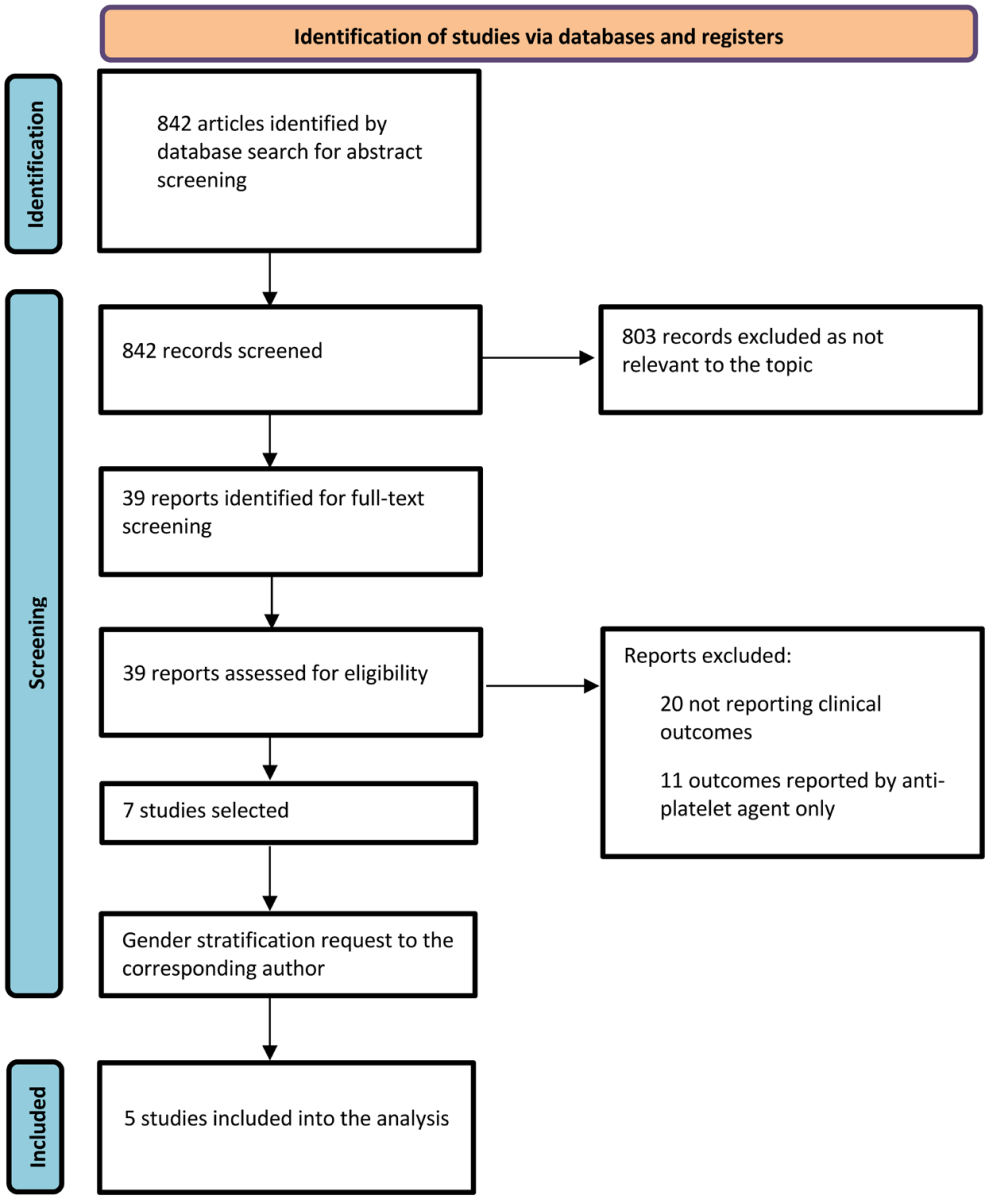
Flow diagram of the search for studies included in the meta‐analysis according to the Preferred Reporting Items for Systematic Review and Meta‐Analyses statement (PRISMA).

The data supporting the results of this study are available from the corresponding author on request. A systematic literature review according to the Preferred Reporting Items for Systematic Reviews and Meta‐Analyses (PRISMA) guidelines was conducted as described previously (Figure 1).^6^ In short, several databases were searched by two independent reviewers using the following search terms: “platelet activity,” “IPF,” “reticulated platelet,” “platelet turnover,” “immature platelet,” “immature platelet fraction,” “immature platelet count,” “cardiovascular death,” “cardiovascular event,” “cardiovascular disease,” “CAD,” “coronary artery disease,” “percutaneous coronary intervention,” “PCI,” “acute coronary syndrome,” “revascularization,” “bleeding,” “stroke”. We imposed no restrictions on language, publication date or status to ensure a comprehensive inclusion of relevant studies. References from selected studies, as well as relevant systematic reviews, meta‐analyses, and editorials, were also reviewed for additional sources. This systematic analysis was registered on PROSPERO with the following registration number: CRD42022381282.

After checking the studies for eligibility and quality, studies reporting clinical outcomes related to high versus low levels of RPs, RPs^high^ and RPs^low^ respectively, focusing specifically on patients with acute coronary syndrome (ACS) or chronic coronary syndrome (CCS) were included in the analysis.^6^ Studies were excluded if they compared outcomes based on non‐RP platelet parameters, lacked reported clinical outcome data, or had overlapping patient populations.^6^ In order to assess and analyse sex‐specific data, corresponding authors of each study were contacted and kindly provided their patient‐level data for further analysis. Two studies were excluded due to data unavailability. Data was extracted and standardized in a uniform database. Risk‐of‐bias assessment was performed using the Risk Of Bias In Non‐randomized Studies of Interventions (ROBINS‐I) tool assessing seven domains of bias for each outcome: confounding, selection of participants into the study, classification of interventions, deviation from intended interventions, missing data, measurement of outcomes, and selection of the reported results.^10^

### 2.2 Endpoint Measures

The primary endpoint of this study was MACCE, based on each trial's specific definition of the primary ischemic outcome (Table 1). Secondary endpoints included cardiovascular death (CVD), myocardial infarction (MI), stroke, urgent coronary revascularization, and any bleeding events. Endpoints were assigned according to the definitions and timelines used in each respective study.

**TABLE 1:** Design of the studies and patients' characteristics.

	Cesari et al. (2013)	Freynhofer et al. (2017)	Perl et al. (2019)	Tscharre et al. (2019)	Bongiovanni et al. (2022)
	April 2008 to April 2009	May 2009 to December 2010	June 2016 to February 2017	May 2009 to December 2010	September 2013 to February 2018‐
Country	Italy	Austria	Israel	Austria	Multicentric: two german centers
Enrolment strategy	Consecutive patients	Consecutive patients	Consecutive patients	Consecutive patients	RCT pre‐specified substudy
Participants (*n*)	229 patients	486 patients	96 patients	477 patients	577 patients
Female (*n*, %)	75 (32.8%)	154 (31.7%)	24 (25.0%)	149 (31.2%)	125 (21.7%)
Type of MI/Type of cases	ACS	CCS and ACS	CCS with DM	CCS and ACS	ACS
Method of RP measurement	Sysmex XE‐2100 haematology analyser	Sysmex XE‐2100 haematology analyser	Sysmex XE‐2100 haematology analyser	Sysmex XE‐2100 haematology analyser	Sysmex XE‐5000 device and Sysmex XN device
RPS laboratory parameter tested	IPF%, H‐IPF	IPF%	IPF%	IPC	IPF%, H‐IPF
Blood sampling	24–48 h after PCI	6–24 h after index PCI	During routine follow‐up visit	6–24 hours after PCI	At admission and within 48 hours after randomization
Median follow‐up	1 year	190 days (IQR 180–243)	2 years	5.8 years (IQR 4.2–6.5)	1 year
Primary ischemic outcome	Cardiovascular deaths: defined as death in the presence of ACS, cardiac arrhythmias or HF	MACCE: composite of all‐cause or cardiovascular death, MI, unplanned revascularization, ST, UA, TIA or stroke)	MACE: death, MI, cerebrovascular accident and urgent revascularization	MACE: composite of cardiovascular death, MI or stroke	Composite of death, myocardial infarction, or stroke
Cut‐off values for RPs^high^	IPF ≥3.3% and H‐IPF ≥.9% Cut‐off based on ROC analyses	IPF% ≥ 3.35% Cut‐off based on ROC analyses	IPF% > median	IPC ≥7.600/μL (median)	IPF% ≥ 3.6% (median)
Primary ischemic outcome in RPs^high^ vs. RP^low^	–	14.4% vs. 7.4% *p* = .014	22.6% vs. 5.6% *p* = .011	–	13.0% (37/284) vs. 7.2% (21/293)
Adjusted ischemic endpoint risk for RPs^high^	IPF ≥3.3%: OR 4.15 (95% CI 1.24–13.91), *p* = .02 H‐IPF ≥.9%: OR 5.03 (95% CI 1.38–18.38), *p* = .01	IPF ≥3.35%: OR 1.14 (95% CI, 1.001–1.288), *p* = .048	IPF% > median: OR 1.97 (95% CI, 1.1128–3.432), *p* = .017	IPC ≥ 7.600/μL: HR 1.72 (95% CI, 1.152–2.559), *p* = .007	IPF ≥ 3.6%: HR 1.74 (95% CI, 1.02–3.00), *p* = .044
Bleeding events definition	–		BARC‐defined bleeding events (type 1 to 5)	TIMI criteria (Major and Minor)	BARC‐defined bleeding events (only type 3 to 5)
Bleeding events rates in RPs^high^ vs. RP^low^	–	–	2.2% vs. 24.1% (*p* = .001)	5.5% vs. 9.2% (*p* = .117)	5.8% vs. 9.1%
Adjusted Bleeding risk for RPs^high^	–		OR .13 (95% CI .03–.63)	OR .57 (95% .28; 1,16)	OR 1.64 (95% CI, .87,3.09)

### 2.3 Statistics

We performed a meta‐analysis to evaluate the primary and secondary endpoints based on RPs levels, stratified by sex. Populations were dichotomized into high and low RPs groups as defined in each study. A random‐effects model was employed to compute pooled odds ratios (OR) and 95% confidence intervals using the Hartung‐Knapp‐Sidik‐Jonkman method.^11^ To assess sex‐specific differences, subgroup analyses were conducted using a Chi‐squared test to determine whether the odds ratios between the males and females were significantly different. For each secondary endpoint, studies with 0 events in either the treatment or control group, a continuity correction of .5 was applied to stabilize OR estimates. Heterogeneity was assessed using Cochran's *Q* test, the *I* ^2^ statistic. Sensitivity analysis was performed using a leave‐one‐out approach for the primary endpoint to assess the robustness of the results. All analyses were performed using Stata version 18.0 (Statistical software for data science, StataCorp).

## 3 RESULTS

A total of 5 studies were included in the final analysis^6^, ^12^, ^13^, ^14^, ^15^ (Figure 1), comprising 1835 patients (527 females and 1308 males). The design and patient characteristics of the included studies are reported in Table 1. Included studies were conducted between 2008 and 2018. Two studies included both ACS and CCS patients,^13^, ^15^ one only CCS^14^ and two only ACS.^6^, ^12^ Three studies set the cut‐off to define patients with increased RPs (RPs^high^) based on the median, while two studies used ROC analysis (Table 1). Details on study biases are provided in Table S1.

### 3.1 Primary endpoint

**FIGURE 2:**
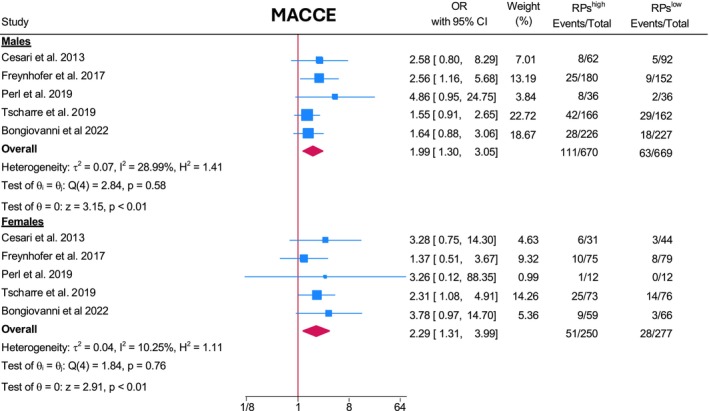
MACCE. Reticulated platelets were predictive for MACCE in both sexes. No significant difference between sexes was observed (test for group difference *p* = .70). CI, Confidence interval; MACCE, Major adverse cardiovascular and cerebrovascular events; OR, Odds ratio; Heterogeneity: Cochran's Q test and the *I* ^2^ statistic.

Reticulated platelets were predictive for MACCE in both sexes, (males: OR 1.99, 95% CI [1.3, 3.05] *I* ^2^ = 28.99%; females: OR 2.29, 95% CI [1.31, 3.99] *I* ^2^ = 10.25%; Figure 2). No significant difference between sexes was observed (test for group difference *p* = .70). Leave‐one‐out analysis is provided in the Figures S1 and S2.

### 3.2 Secondary endpoints

CVD was reported in all 5 studies.^6^, ^12^, ^13^, ^14^, ^15^ MI was reported in 5 studies; however, 1 study had to be excluded for males as they did not report any event. Stroke was reported in 5 studies; however, 3 studies needed to be excluded for females as they did not report any events.

**FIGURE 3:**
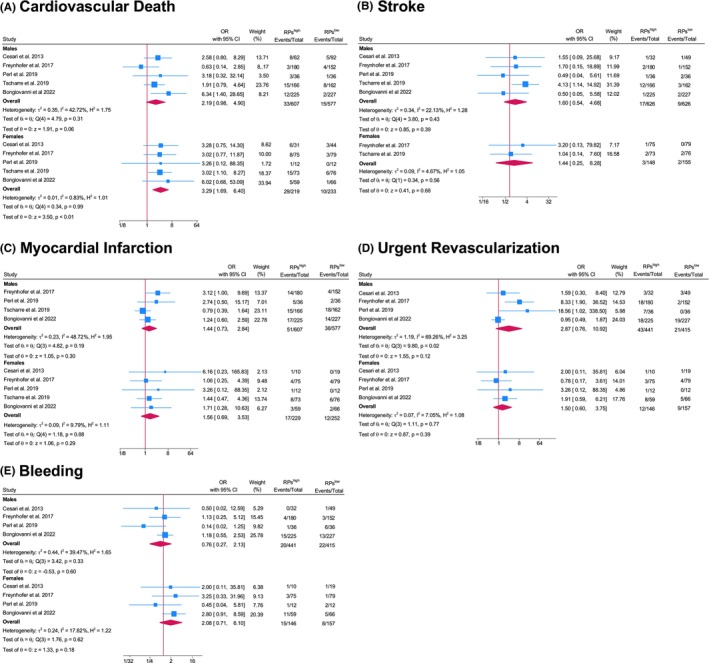
Secondary endpoints: Cardiovascular death, myocardial infarction, stroke, urgent coronary revascularization, and any bleeding events. CI, Confidence interval; OR, Odds ratio, Heterogeneity: Cochran's Q test and the *I* ^2^ statistics.

We detected a significant association of RPs^high^ with CVD in females (OR 3.29 95% CI [1.69, 6.40] *I* ^2^ = .83%) but not in males (OR 2.19, 95% CI [.98, 4.90] *I* ^2^ = 42.72%; Figure 3). However, the results concerning males were close to reaching significance (*p* = .06) and the test for group differences between sexes was not significant (*p* = .48). We did not detect any significant associations of RPs^high^ with the other components of MACCE for both males and females (MI males: OR 1.44, 95% CI [.73, 2.84] *I* ^2^ = 48.72%; females: OR 1.56 95% CI [.69, 3.53] *I* ^2^ = 9.79%) and stroke (males OR 1.60, 95% CI [.54, 4.68] *I* ^2^ = 22.13%: females OR 1.44 95% CI [.25, 8.28] *I* ^2^ = 4.67%) (Figure 3). Also, RPs^high^ did not show a significant association with urgent revascularization (males: OR 2.87 95% CI [.76, 10.92], *I* ^2^ = 69.26%: females OR 1.50 [.60, 3.75] *I* ^2^ = 7.05%) (Figure 3). Bleeding was reported in 4 studies.^6^, ^12^, ^13^, ^14^, ^15^ Independently of sex, RPs^high^ was not significantly associated with bleeding complications (males: OR .76 95% CI [.27, 2.13] *I* ^2^ = 39.47%; females: OR 2.08 95% CI [.71, 6.10] *I* ^2^ = 17.82%) (Figure 3). Across all secondary endpoints, no significant sex‐based differences were observed (MI *p* = .98; stroke *p* = .98; urgent revascularization *p* = .35; bleeding *p* = .28).

## 4 DISCUSSION

Our meta‐analysis represents the first comprehensive effort to elucidate the sex‐specific prognostic value of RPs in cardiovascular disease. Given the underrepresentation of females in preclinical and clinical studies and the well known biological and physiological differences between sexes in cardiovascular disease, this study contributes to addressing this critical knowledge gap. The major findings of this work are as follows: (1) RPs^high^ were significantly associated with MACCE in both males and females; (2) RPs^high^ were significantly associated with CVD in females and showed a trend toward significance in males; (3) RPs^high^ were not associated with all other secondary endpoints including bleeding, MI, stroke and urgent revascularization, irrespective of sex.

These results are consistent with our previously performed meta‐analysis, which observed a significant association of RPs^high^ with MACCE (167% increased risk) and CVD (109% increased risk).^6^ While the earlier aggregate‐data meta‐analysis by Bongiovanni et al. demonstrated the overall prognostic value of RPs in coronary artery disease, the current work builds upon that foundation by incorporating patient‐level, sex‐stratified data to examine whether the predictive utility of RPs differs between males and females. This patient‐level sex subanalysis further highlights a stronger association between RPs^high^ and CVD in females (229% increased risk) compared to males (119% increased risk), although the latter narrowly missed statistical significance. Although these results are influenced by the limited number of included studies—which may explain why the increased risk in males did not reach statistical significance—the higher rate of cardiovascular death observed in females appears robust enough to overcome their underrepresentation. This finding highlights the need for further prospective investigations to validate and explore the underlying mechanisms driving these sex‐specific differences.

Prior research has clarified that in addition to the recognized variation in symptoms and presentations of coronary syndromes between sexes, women's smaller epicardial coronary arteries compared to men's, along with disparities in shear stress and the presence of inflammatory mediators throughout life, might influence the progression of coronary artery disease (CAD).^16^ However, these mechanisms remain speculative and further investigation of the pathophysiological reasons for the well‐known sex differences in cardiovascular disease is warranted.^9^

In this meta‐analysis, RPs^high^ were significantly associated with MACCE in both male and female. Our findings suggest that the biological mechanisms by which RPs contribute to thrombogenesis and cardiovascular events may operate similarly across sexes, but they could lead to different outcomes as suggested by the CVD results.

While the prothrombotic features of RPs have been well documented at both transcriptomic and proteomic levels, no preclinical study to date has specifically investigated biological differences in RPs across sexes. Existing evidence demonstrates that RPs exhibit heightened prothrombotic activity, including the upregulation of specific transmembrane receptors and activation pathways, such as the PAR1/4 thrombin and GPVI collagen pathways.^6^, ^17^ These features suggest that RPs may play a disproportionately significant role in promoting thrombosis. In females, the known protective effects of hormone‐related biology—such as oestrogen‐mediated vascular protection and modulation of platelet function—might initially mitigate cardiovascular risks.^18^, ^19^, ^20^, ^21^, ^22^ However, the heightened prothrombotic potential of RPs could potentially override these protective mechanisms in patients with elevated RPs, particularly under pathological conditions, contributing to increased cardiovascular mortality.

Emerging hypotheses suggest that sex hormones, particularly oestrogen, might play a role in modulating vascular and platelet function.^18^ Oestrogen possibly exerts vasodilatory, antioxidant, and anti‐inflammatory effects, partly through the upregulation of nitric oxide production and suppression of reactive oxygen species.^23^ However, these protective effects decline significantly after menopause, which could lead to endothelial dysfunction characterised by a pro‐inflammatory and pro‐thrombotic state and thereby increasing the risk of cardiovascular events.^24^

In addition, platelets in women possibly exhibit heightened responsiveness to agonists such as thrombin and ADP along with increased surface expression of activation markers and greater reactivity overall.^20^, ^25^ Reticulated platelets, due to their RNA‐rich, immature phenotype, may amplify these differences, resulting in stronger platelet activation cascades in females. Moreover, age‐related changes in platelet biology appear to be more pronounced in women than in men, suggesting that female platelets show greater age‐associated functional alterations.^20^ These biological differences may contribute to the higher predictive value of RPs for CVD in women.

In general, the prognostic relevance of platelet‐related biomarkers is highlighted by a recent meta‐analysis demonstrating that elevated admission MPV—a surrogate of platelet turnover and indirectly related RPs—is associated with increased long‐term mortality in patients with CAD, including those with ACS.^26^

Nevertheless, the interplay between RPs' prothrombotic activity and sex‐specific physiological factors remains speculative and highlights the urgent need for targeted preclinical studies. Investigating how sex‐based differences influence RP biology at molecular, cellular, and functional levels could provide valuable insights into their contribution to cardiovascular events and mortality, eventually leading to personalised therapies.

## 5 LIMITATIONS

This meta‐analysis has several limitations. One notable limitation is the clinical and methodological heterogeneity among the included studies, which may impact the interpretation and generalizability of our findings. Although all studies utilized the Sysmex platform to measure RPs, the specific parameters used varied: four studies stratified patients based on the immature platelet fraction (IPF), while one used the immature platelet count (IPC). Additionally, there were inconsistencies in the definition of high RPs (RPs^high^): three studies employed median values and two relied on receiver operating characteristic (ROC) curve analysis. These differences in biomarker definition and classification could introduce variability in effect estimates and complicate direct comparisons across studies. Moreover, variations in patient populations (e.g., acute vs. chronic coronary syndromes), study design, and endpoint definitions further contribute to heterogeneity, potentially influencing the pooled results. In addition, the overall number of included studies was small, and the limited representation of female patients across datasets remains a major constraint. Further, despite our efforts to include sex‐specific data, the persistent underrepresentation of women in cardiovascular research may have limited our ability to detect subtle sex differences. Additionally, our analysis was constrained to five studies, reducing statistical power, especially for secondary endpoints. Finally, the absence of comprehensive patient‐level data prevented detailed stratified analyses based on additional baseline characteristics.

## 6 CONCLUSION

Elevated RPs are a robust prognostic biomarker for MACCE in coronary artery disease, irrespective of sex. However, the stronger association between RPs^high^ and CVD in females highlights the need for further investigation into sex‐specific mechanisms of platelet biology. Addressing the underrepresentation of women in cardiovascular research is critical to refining our understanding of sex‐specific risks and optimising clinical care.

## AUTHOR CONTRIBUTIONS

SE, SGK: writing of the manuscript, data analysis, statistical analysis, data interpretation. AP: statistical analysis. MT, MF, LP, RK, FC, RM, LN, IB: data acquisition, critical review of the manuscript. PWR: data interpretation, critical review of the manuscript. MC: data analysis, data interpretation, critical review of the manuscript. DB: concept of the study, writing of the manuscript, statistical analysis, data interpretation. All authors agreed to the final version of the manuscript. SE, SGK: equal contribution, shared first authorship.

## FUNDING INFORMATION

None.

## CONFLICT OF INTEREST STATEMENT

All authors have nothing to declare.

## Supporting information


Appendix S1.

## Data Availability

Primary data are available upon request (contact: see corresponding author).
