# Supplementary material for: Sex‐specific predictive value of reticulated platelets in coronary artery disease: A systematic review and meta‐analysis
Source: Eur J Clin Invest. 2025 May 19;55(10):e70078. doi: 10.1111/eci.70078 (PMC12434453; doi:10.1111/eci.70078)
Supplement: Supplementary file 1 — Appendix S1. [file ECI-55-e70078-s001.zip › eci70078-sup-0001-TableS1@Supplemental file.docx]

**Sex-Specific Predictive Value of Reticulated Platelets in Coronary Artery Disease: A Systematic Review and Meta-Analysis**

Sex Differences in Reticulated Platelets as Predictors of Cardiovascular Risk

**SUPPLEMENTAL FILE**

Sebastien Elvinger ¹^,*^, Stephanie G. Kuehne ¹^,*^, Andrea Patrignani², Maximilian Tscharre^3,4^ , Matthias Freynhofer^5^, Leor Perl^6^ , Ran Kornowski^6^ , Francesca Cesari^7,8^ , Rossella Marcucci^7,8^, Laura Novelli^2^, Isabell Bernlochner^9^, Philip W. Raake¹, Mauro Chiarito^2^, Dario Bongiovanni^1^

¹ Department of Internal Medicine I, Cardiology, University Hospital Augsburg, University of Augsburg, Germany

² Department of Cardiovascular Medicine, Humanitas Clinical and Research Center IRCCS and Humanitas University, Rozzano, Milan, Italy

^3^ Department of Internal Medicine with Cardiology, Nephrology and Intensive Care Medicine, Universitätsklinikum Wiener Neustadt, Austria.

^4^ Department of Medicine, Faculty of Medicine and Dentistry, Danube Private University, Krems, Austria

^5^ 3rd Medical Department, Cardiology and Intensive Care Medicine, Clinic Ottakring, Vienna, Austria

^6^ The Cardiovascular Division, Beilinson Hospital, Rabin Medical Center, Petach Tikva and the Faculty of Medical and Health Sciences, Tel Aviv University, Tel Aviv, Israel.

^7^ Department of Experimental and Clinical Medicine, University of Florence, Florence, Italy.

^8^ Atherothrombotic Diseases Centre, Careggi University Hospital, Florence,Italy.

^9^ Department of Internal Medicine I, School of Medicine, University Hospital rechts der Isar, Munich, Germany

Address for correspondence:

| PD Dr. med. Dario Bongiovanni, PhD  Department of Internal Medicine I, Cardiology  University Hospital Augsburg  University of Augsburg  Stenglinstraße 2, 86156 Augsburg, Germany  Tel.: 0821-400-2355  E-Mail: dario.bongiovanni@uk-augsburg.de | Prof. Dr. med. Philip Raake  Department of Internal Medicine I, Cardiology  University Hospital Augsburg  University of Augsburg  Stenglinstraße 2, 86156 Augsburg, Germany  Tel.: 0821-400-2355  E-Mail: philip.raake@uk-augsburg.de |
| --- | --- |

**Supplemental Figure 1: Women Leave-one-out analysis primary endpoint (MACCE)**


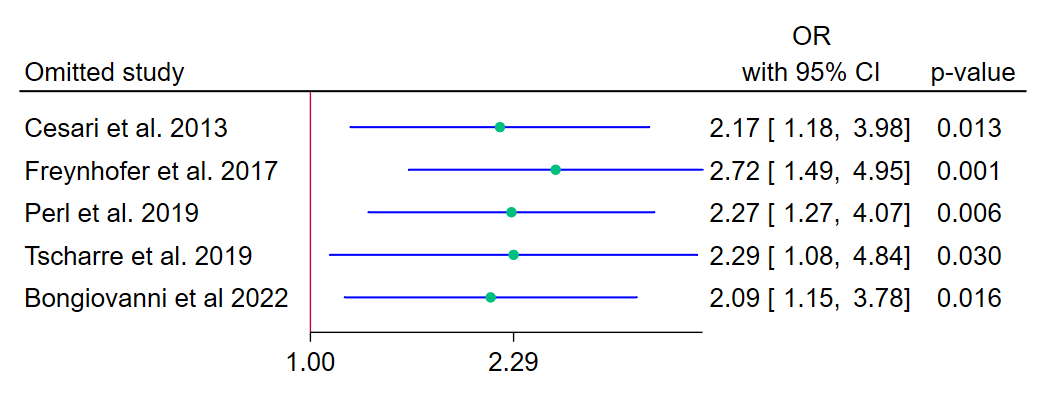


**Supplemental Figure 1 legend:** The Leave-One-Out Analysis demonstrates that the results remain consistently significant and the ORs are similar, regardless of the excluded study, confirming the robustness of the overall analysis.

**Supplemental Figure 2: Men Leave-one-out analysis primary endpoint (MACCE)**


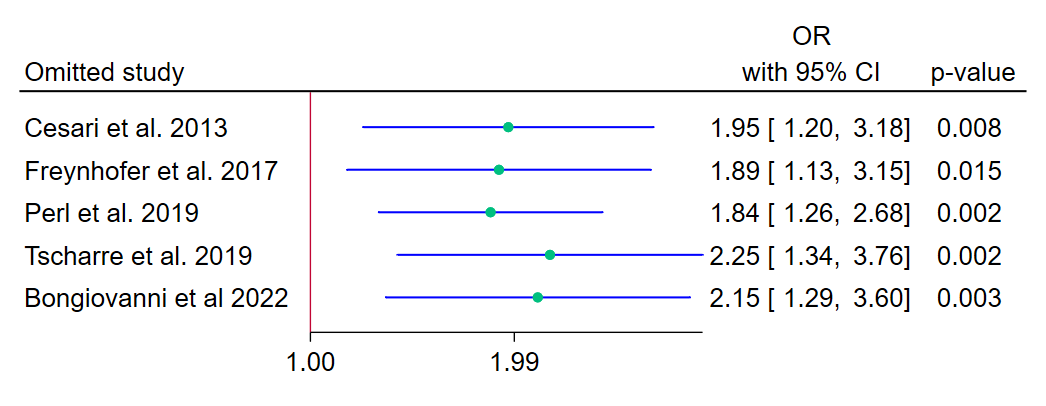


**Supplemental Figure 2.** The Leave-One-Out Analysis demonstrates that the results remain consistently significant and the ORs are similar, regardless of the excluded study, confirming the robustness of the overall analysis.

**Supplementary Table 1: Risk of Bias assessment**


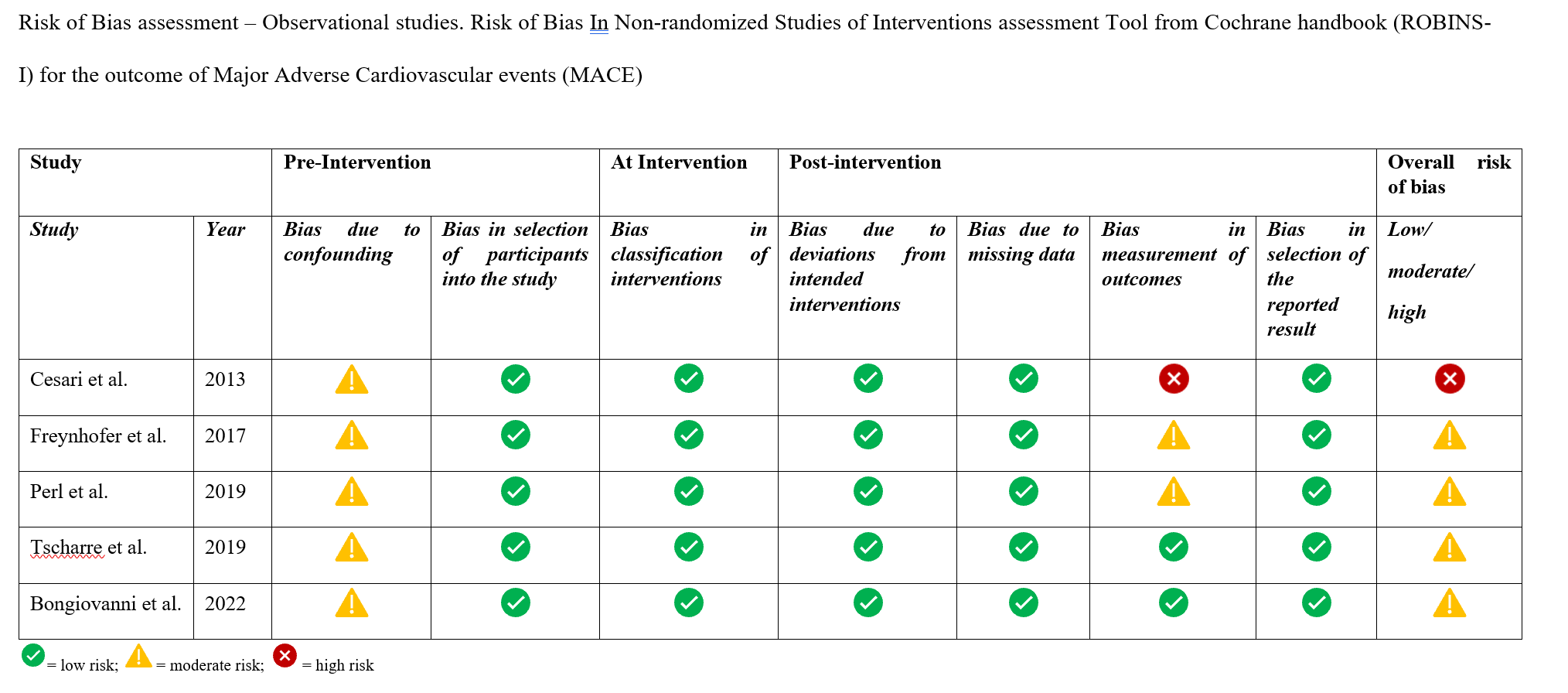


Risk of Bias assessment – Observational studies. Risk of Bias In Non-randomized Studies of Interventions assessment Tool from Cochrane handbook (ROBINS-I) for the outcome of Major Adverse Cardiovascular events (MACE)
