# Supplementary material for: Sex‐specific predictive value of reticulated platelets in coronary artery disease: A systematic review and meta‐analysis
Source: Eur J Clin Invest. 2025 May 19;55(10):e70078. doi: 10.1111/eci.70078 (PMC12434453; doi:10.1111/eci.70078)
Supplement: Supplementary file 1 — Appendix S1. [file ECI-55-e70078-s001.zip › eci70078-sup-0002-Supinfo1@Supplemental table 1 ROBINS-I_Sex.docx]

Risk of Bias assessment – Observational studies. Risk of Bias In Non-randomized Studies of Interventions assessment Tool from Cochrane handbook (ROBINS-I) for the outcome of Major Adverse Cardiovascular events (MACE)

| **Study** | | **Pre-Intervention** | | **At Intervention** | **Post-intervention** | | | | **Overall risk of bias** |
| --- | --- | --- | --- | --- | --- | --- | --- | --- | --- |
| ***Study*** | ***Year*** | ***Bias due to confounding*** | ***Bias in selection of participants into the study*** | ***Bias in classification of interventions*** | ***Bias due to deviations from intended interventions*** | ***Bias due to missing data*** | ***Bias in measurement of outcomes*** | ***Bias in selection of the reported result*** | ***Low/***  ***moderate/***  ***high*** |
| Cesari et al. | 2013 | 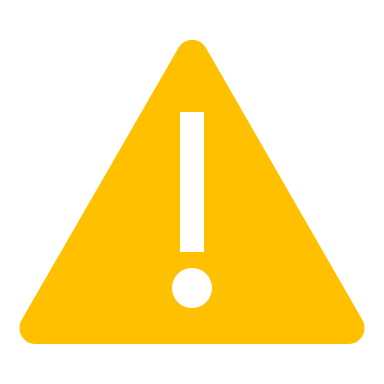 | 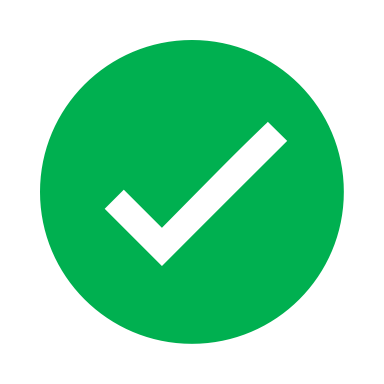 | 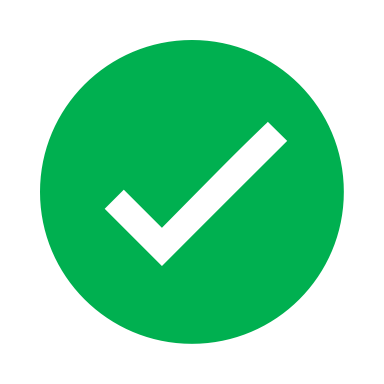 | 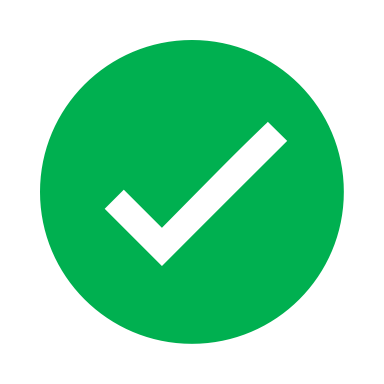 | 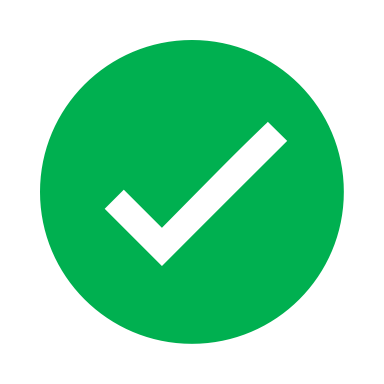 | 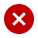 | 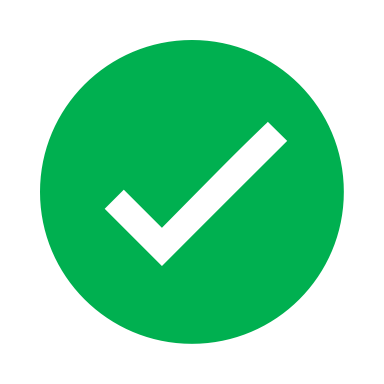 | 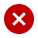 |
| Freynhofer et al. | 2017 | 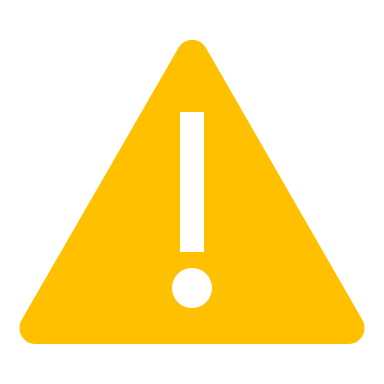 | 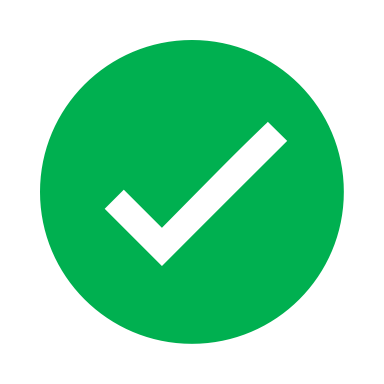 | 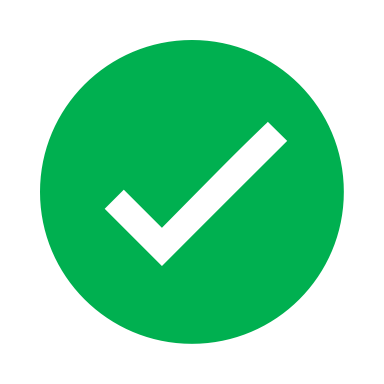 | 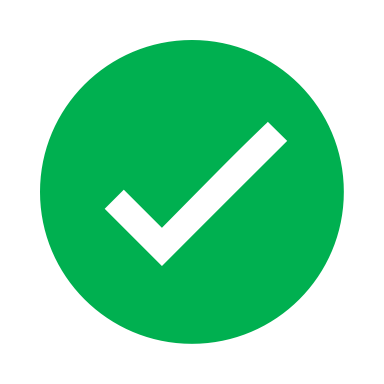 | 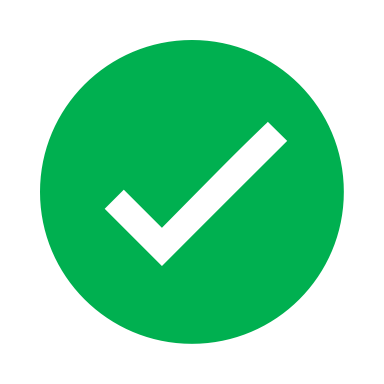 | 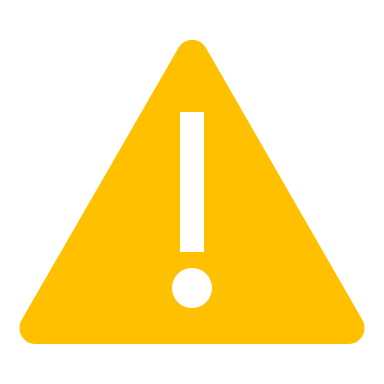 | 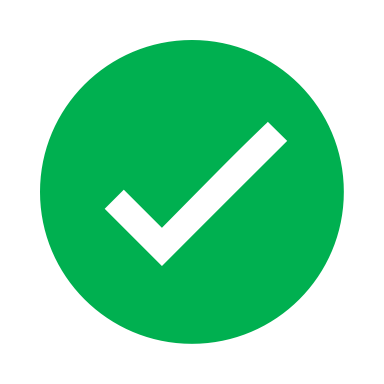 | 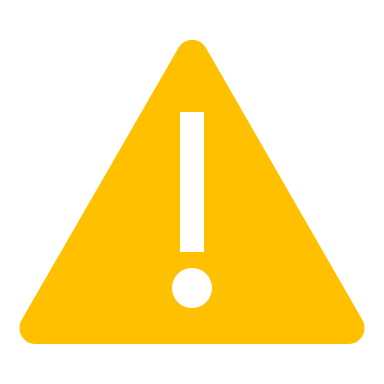 |
| Perl et al. | 2019 | 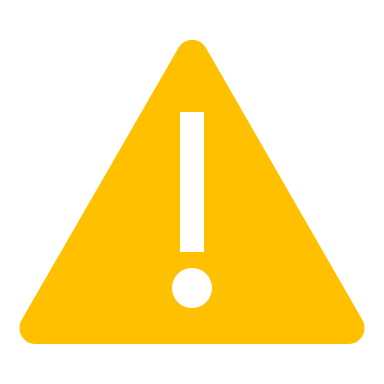 | 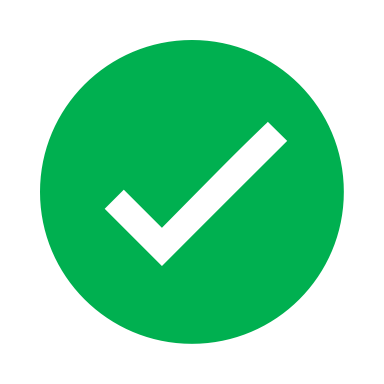 | 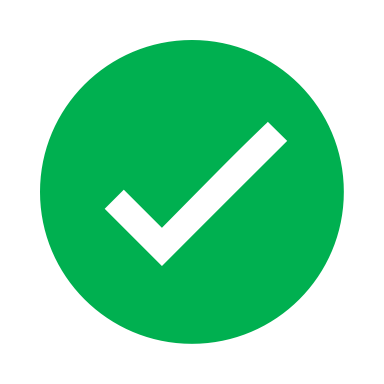 | 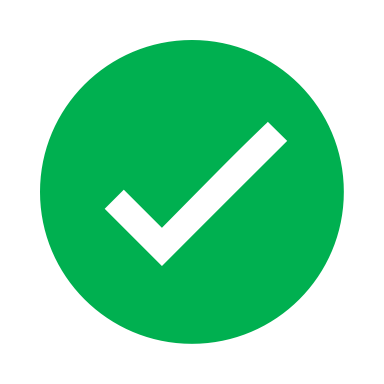 | 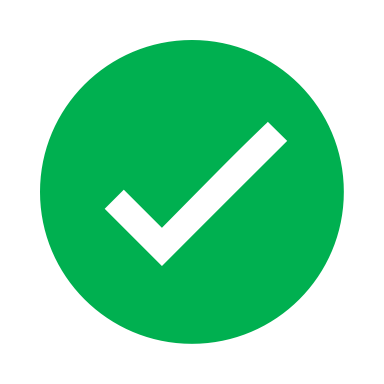 | 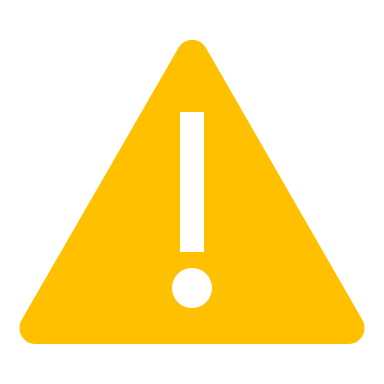 | 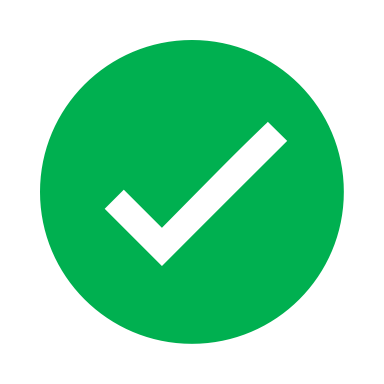 | 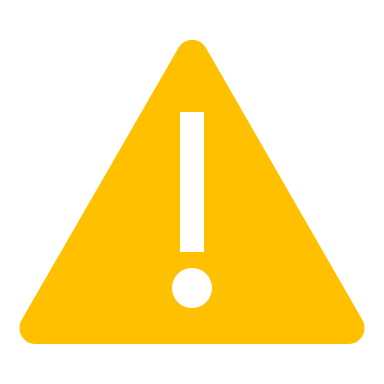 |
| Tscharre et al. | 2019 | 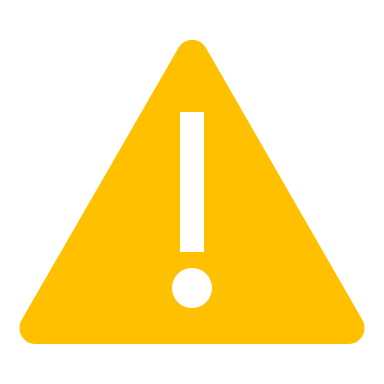 | 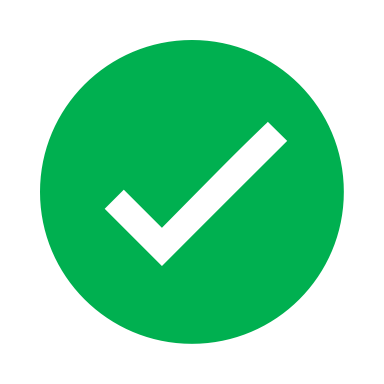 | 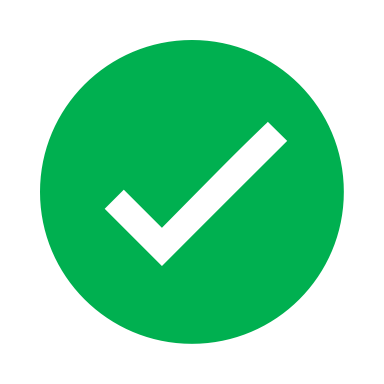 | 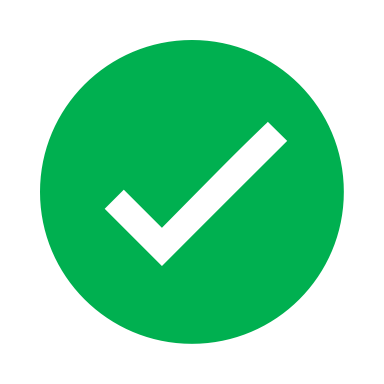 | 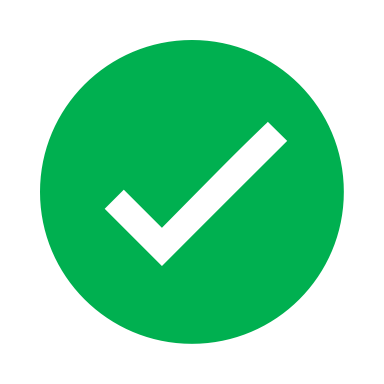 | 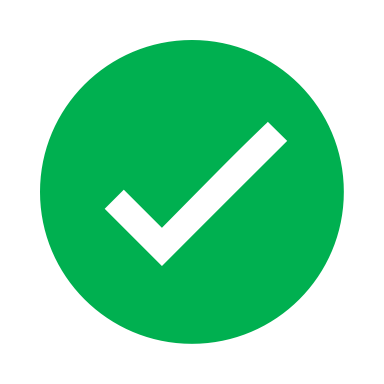 | 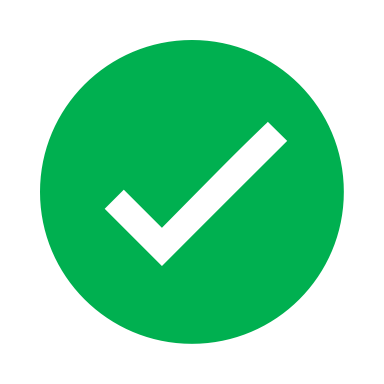 | 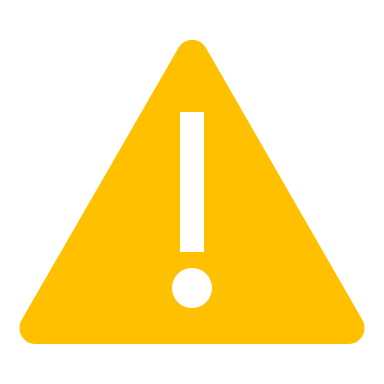 |
| Bongiovanni et al. | 2022 | 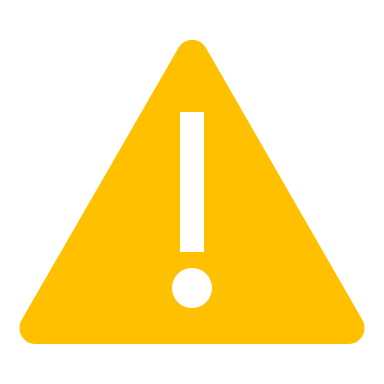 | 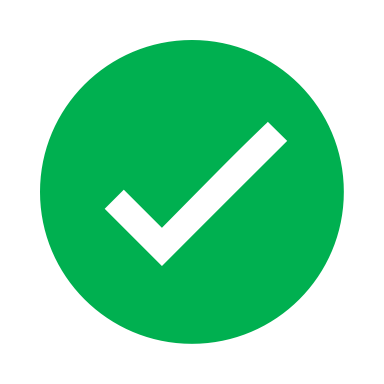 | 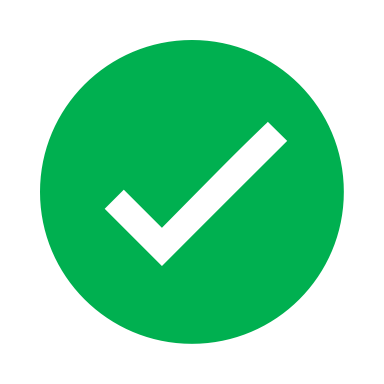 | 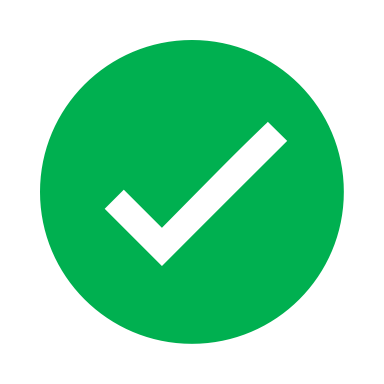 | 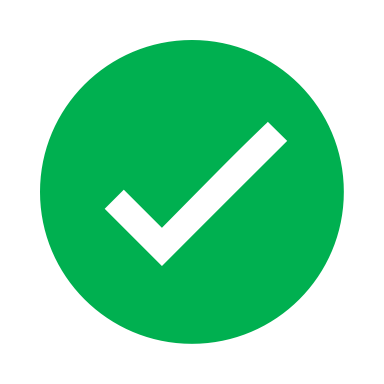 | 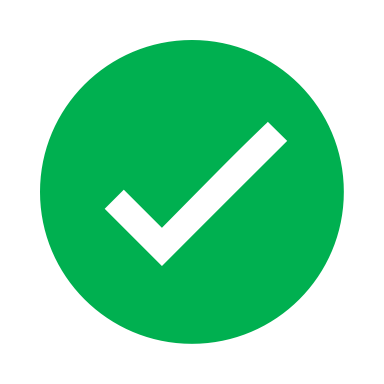 | 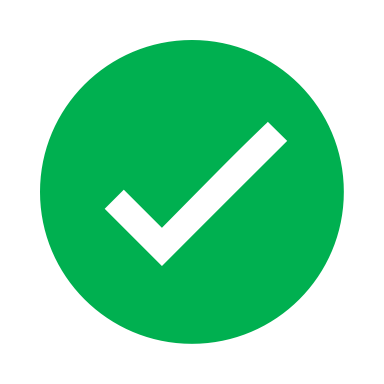 | 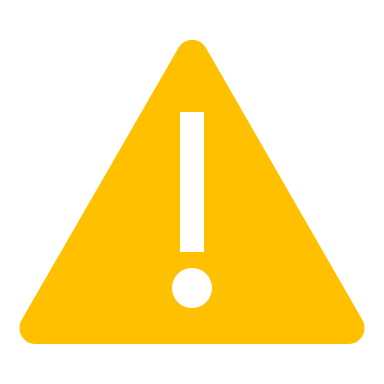 |


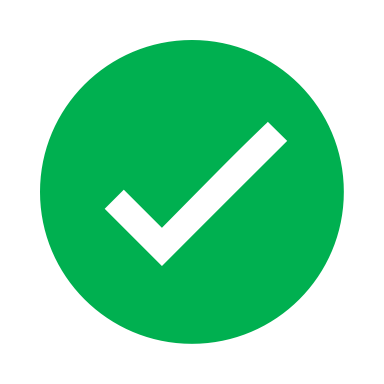
= low risk;
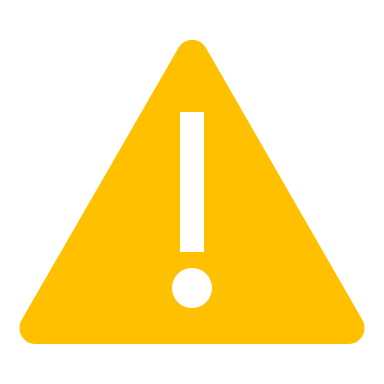
 = moderate risk;
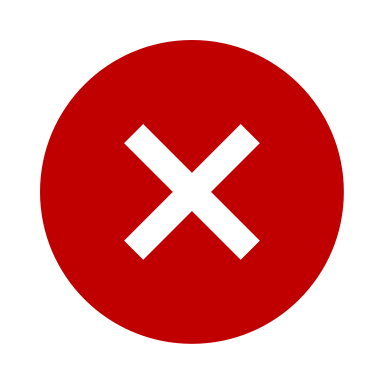
 = high risk
